# Sympathetic Activation Does Not Affect the Cardiac and Respiratory Contribution to the Relationship between Blood Pressure and Pial Artery Pulsation Oscillations in Healthy Subjects

**DOI:** 10.1371/journal.pone.0135751

**Published:** 2015-08-18

**Authors:** Pawel J. Winklewski, Yurii Tkachenko, Kamila Mazur, Jacek Kot, Marcin Gruszecki, Wojciech Guminski, Krzysztof Czuszynski, Jerzy Wtorek, Andrzej F. Frydrychowski

**Affiliations:** 1 Institute of Human Physiology, Medical University of Gdansk, Gdansk, Poland; 2 National Centre for Hyperbaric Medicine, Institute of Maritime and Tropical Medicine, Medical University of Gdansk, Gdynia, Poland; 3 Department of Biomedical Engineering, Faculty of Electronics, Telecommunications and Informatics, Gdansk University of Technology, Gdansk, Poland; 4 Department of Radiology Informatics and Statistics, Medical University of Gdansk, Gdansk, Poland; 5 Department of Computer Communications, Faculty of Electronics, Telecommunications and Informatics, Gdansk University of Technology, Gdansk, Poland; The University of Tokyo, JAPAN

## Abstract

**Introduction:**

Using a novel method called near-infrared transillumination backscattering sounding (NIR-T/BSS) that allows for the non-invasive measurement of pial artery pulsation (cc-TQ) and subarachnoid width (sas-TQ) in humans, we assessed the influence of sympathetic activation on the cardiac and respiratory contribution to blood pressure (BP) cc-TQ oscillations in healthy subjects.

**Methods:**

The pial artery and subarachnoid width response to handgrip (HGT) and cold test (CT) were studied in 20 healthy subjects. The cc-TQ and sas-TQ were measured using NIR-T/BSS; cerebral blood flow velocity (CBFV) was measured using Doppler ultrasound of the left internal carotid artery; heart rate (HR) and beat-to-beat mean BP were recorded using a continuous finger-pulse photoplethysmography; respiratory rate (RR), minute ventilation (MV), end-tidal CO_2_ (EtCO2) and end-tidal O_2_ (EtO2) were measured using a metabolic and spirometry module of the medical monitoring system. Wavelet transform analysis was used to assess the relationship between BP and cc-TQ oscillations.

**Results:**

HGT evoked an increase in BP (+15.9%; P<0.001), HR (14.7; P<0.001), SaO_2_ (+0.5; P<0.001) EtO_2_ (+2.1; P<0.05) RR (+9.2%; P = 0.05) and MV (+15.5%; P<0.001), while sas-TQ was diminished (-8.12%; P<0.001), and a clear trend toward cc-TQ decline was observed (-11.0%; NS). CBFV (+2.9%; NS) and EtCO_2_ (-0.7; NS) did not change during HGT. CT evoked an increase in BP (+7.4%; P<0.001), sas-TQ (+3.5%; P<0.05) and SaO_2_(+0.3%; P<0.05). HR (+2.3%; NS), CBFV (+2.0%; NS), EtO_2_ (-0.7%; NS) and EtCO_2_ (+0.9%; NS) remained unchanged. A trend toward decreased cc-TQ was observed (-5.1%; NS). The sas-TQ response was biphasic with elevation during the first 40 seconds (+8.8% vs. baseline; P<0.001) and subsequent decline (+4.1% vs. baseline; P<0.05). No change with respect to wavelet coherence and wavelet phase coherence was found between the BP and cc-TQ oscillations.

**Conclusions:**

Short sympathetic activation does not affect the cardiac and respiratory contribution to the relationship between BP—cc-TQ oscillations. HGT and CT display divergent effects on the width of the subarachnoid space, an indirect marker of changes in intracranial pressure.

## Introduction

The cardiovascular system consists of the heart and blood vessels. The heart can be described as a pump that drives the blood through the closed circuit of elastic vessels. The respiratory activity generates a pressure that assists in the return of blood to the heart. The flow of blood largely depends on the resistance of the vessels, which is controlled by adjustment of their diameter. Consequently, the power of cardiac oscillations dominates the aortic flow and is significantly decreased in blood flow through the capillaries. The cardiac and respiratory oscillations have frequencies of around 1 Hz and 0.3 Hz, respectively, which originate centrally and are propagated through the system [1]. Within the brain, the pial artery carry a significant amount of the vascular resistance, making them important regulators of cerebral blood flow (CBF) [2]. Current thinking about the regulation of CBF is dominated by the dynamic cerebral autoregulation model where cerebral autoregulation is considered a high-pass filter (for review, see [3]). The dominant role of cerebral autoregulation is, however, increasingly being challenged by several authors who have indicated that CBF is also modulated by several other factors, including elastic vessel mechanical (Windkessel) properties or cardiac compensatory mechanisms [4–9]. Such mechanisms may also operate at higher frequencies than the typical autoregulatory range.

Non-invasive assessment of pial artery pulsation became possible due to a recently developed method based on infrared radiation (IR) called near-infrared transillumination/backscattering sounding (NIR-T/BSS). In contrast to near-infrared spectroscopy (NIRS), which relies on the absorption of IR by haemoglobin [10], NIR-T/BSS uses the subarachnoid space (SAS), which is filled with translucent cerebrospinal fluid, as a propagation duct for IR [11]. Thus, NIR-T/BSS enables the assessment of instantaneous changes in the SAS width in humans (sas-TQ). Fast oscillations in the width of the SAS—referred to as the cardiac component of subarachnoid width pulsation (cc-TQ)–result from heart-generated pial artery pulsation. The NIR-T/BSS high sampling frequency (70 Hz) allows for the signal analysis up to 5 Hz. The power spectrum density of cc-TQ shows clear peaks at the fundamental frequency (f_0_) and its harmonics (f_1_, f_2_, f_3_) [12]. As shown previously, changes in the SAS width correlate with intracranial pressure to a considerable extent, providing sufficient evidence of changes in intracranial pressure by measurements of sas-TQ [13,14].

Dynamic cardiovascular responses arise from different autonomic pathways, depending upon the stimuli. A voluntary muscle contraction during the handgrip test (HGT) elicits heart rate (HR) increases through the integration of autonomic regulatory networks with sensory and motor components of the cortex, cerebellum and basal ganglia [15]. Exposure to cold induces autonomic responses through the medullary, hypothalamic and insular cortex areas [15]. A pain stimulus associated with the cold test (CT) may additionally trigger sympathetic action through the integration of sensory input within the medullary, mid-brain, thalamic and insular cortex regions [16,17].

It has previously been shown that breath-hold apnoea diminishes the cardiac contribution to BP cc-TQ oscillations [18]. However, apnoea is associated with hypoxia, hypercapnia and co-activation of both branches of the autonomic nervous system (for review, see [19,20]), and for these reasons cannot be used as a strict model of the sympathetic nervous system (SNS) activation. Furthermore, during apnoea, respiratory activity is clearly absent. Therefore, using two relatively easy-to-perform physiological tests, HGT and CT, we assessed the influence of SNS on the cardiac and respiratory contribution to the relationship between BP cc-TQ oscillations. We hypothesised that both HGT and CT would not affect the cardiac and respiratory contribution to the relationship between BP cc-TQ oscillations, regardless of the fact that the stimuli evoked by the tests are likely transmitted by different central SNS circuits.

## Materials and Methods

### Subjects

Experiments were performed on a group of 20 healthy volunteers (6 females; age 28.5±7.5 years; BMI = 24.2±3.6 kg/m^2^); none of them were smokers. All subjects received detailed information about the study objectives and any potential adverse reactions, and they provided written informed consent to participate in the study. The experimental protocol and the study were approved by the Ethics Committee of the Medical University of Gdansk. Although none of the participants suffered from known disorders or were taking any medication, a general and neurological examination was performed before the experiment. Nicotine, coffee, tea, cocoa and methylxanthine-containing food and beverages were not permitted for 8 hours before the tests. Additionally, prior to each test, the volunteers were asked to rest comfortably for 30 minutes in the supine position.

### Experimental design

All tests were conducted in a comfortable quiet room with a comfortable temperature. The sequence of challenges was HGT followed by CT. For the HGT challenge, subjects were instructed to squeeze an electronic HGT dynamometer held in the right hand at maximum force. They were initially directed to briefly squeeze at maximum effort as a reference. The challenge consisted of a 2-minute strain (indicated by oral communication from the investigator) at 30% of maximum. After practice, subjects were allowed to return to a baseline state. The CT consisted of 10 minutes at baseline, 2 minutes' hand immersion to approximately wrist height in cold water (4°C, verified by digital thermometer) and 10 minutes' recovery. One investigator lifted the hand into and out of the water at the appropriate times.

### Measurements

ECG was recorded using a standard electrocardiograph. Mean BP was measured using continuous finger-pulse photoplethysmography (CNAP, CNSystems Medizintechnik AG, Graz, Austria). Finger blood pressure was calibrated against brachial arterial pressure. Oxyhaemoglobin saturation (SaO_2_) was measured continuously (Massimo Oximeter, Massimo, Milan, Italy) with a finger-clip sensor. Expired air was analysed with a spirometry module of the medical monitoring system (Datex-Ohmeda, GE Healthcare, Wauwatosa, WI, US) for respiratory rate (RR) and minute ventilation (MV). Gas samples from the mouthpiece were constantly analysed using the side-stream technique for end-tidal CO_2_ (EtCO_2_) and end-tidal O_2_ (EtO_2_) with the metabolic module of the same medical monitoring system (Datex-Ohmeda, GE Healthcare, Wauwatosa, WI, US). Doppler ultrasound of the internal carotid artery was performed (*Vivid 7*, GE Healthcare; Little Chalfont, UK) to assess the mean cerebral blood flow velocity (CBFV). Changes in the amplitude of pial artery pulsation and in the width of the SAS with NIR-T/BSS were recorded with a head-mounted SAS 100 Monitor (NIRT sp. z o.o., Wierzbice, Poland). The theoretical and practical foundations of the NIR-T/BSS method have been published previously [11,12]. All variables were recorded continuously or videotaped, and the signals were digitally saved on the computer for further analyses.

### Wavelet analysis

**Fig 1:**
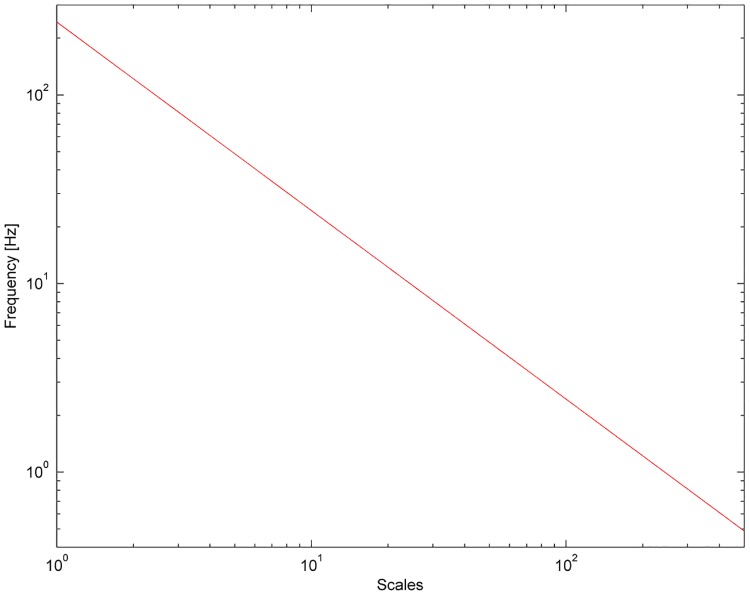
Transition from scales used in wavelet transform analysis to frequency values.

The wavelet transform is a method that transforms a time signal from the time domain to the time-frequency domain. The definition of the wavelet transform is:

$$W\left(s,t\right)=\frac{1}{\sqrt{s}}\int_{-\infty }^{+\infty }\varphi \left(\frac{u-t}{s}\right)g\left(u\right)du,$$ (1)

where *W*(*s*,*t*) is the wavelet coefficient, *g*(*u*) is the time series, and *φ* is the Morlet mother wavelet, scaled by factor *s* and translated in time by *t*. The Morlet mother wavelet is defined by the equation:

$$\varphi \left(u\right)=\;\frac{1}{\sqrt[{4}]{\pi }}e^{-i2\pi u}e^{-0.5u^{2}},$$ (2)

where $=\sqrt{-1}$. The reason for using the Morlet wavelet is its good localisation of events in time and frequency due to its Gaussian shape [18,21]. The frequency is inversely proportional to its corresponding scaling factor s (see Fig 1 in log-log scale). The wavelet transform was calculated in the frequency interval from 0.097 to 56.7 Hz.

Wavelet coherence (WCO) and wavelet phase coherence (WPCO) were estimated using the Matlab function ‘wcoher.m’ with ‘morl’ (Morelet function) as a mother wavelet, and a scale from 1 to 500, which corresponds to the frequency interval from 0.097 to 56.7 Hz. A detailed description with clear examples can be found at: http://www.mathworks.com/help/wavelet/examples/wavelet-coherence.html. In all calculations, we estimated the normalised value of WCO:

$${\text{w}}_{12}=\frac{{\text{w}}_{1}{\left(\text{s},\text{t}\right)}^{*}{\text{w}}_{2}\left(\text{s},\text{t}\right)}{\sqrt{{\text{w}}_{1}{\left(\text{s},\text{t}\right)}^{2}}\sqrt{{\text{w}}_{2}{\left(\text{s},\text{t}\right)}^{2}}}$$ (3)

where w_1_(s,t) (w_2_(s,t)) is the wavelet coefficient for the first (the second) signal and * indicates the complex conjugate. We observed stronger coherence when the WCO value increased. The value of WPCO is between 0 and 1. When two oscillations are unrelated, their phase difference continuously changes with time, thus their WPCO approaches zero. If the WPCO is around 1, the two oscillations are related and significant coherence is observed.

### Statistical analysis

Wilcoxon signed-rank test was used to compare the changes in WCO, WPCO, cc-TQ, BP, HR, CBFV, SaO_2_, EtCO_2_, EtO^2^, respiratory rate and MV in response to HGT and CT.

## Results

HGT evoked an increase in BP, HR, SiO_2_, EtO_2_, respiratory rate and MV, while sas-TQ diminished, and a clear trend toward cc-TQ decline was observed, although the cc-TQ change was not statistically significant. CBFV and EtCO_2_ did not change during HGT (Table 1).

**Table 1:** Effects of 120 s HGT on cc-TQ, sas-TQ, BP, HR, CBFV, EtO_2_, EtCO_2_, SaO_2_, respiratory rate and MV. Data presented as mean values and standard deviations (SD). All % changes are calculated with reference to baseline values.

Variable	Baseline	Handgrip test	% Change	P value
sas-TQ left (AU)	6795±1790	6299±1738	- 7.30	0.000
sas-TQ right (AU)	7287±4746	6635±4572	- 8.94	0.000
cc-TQ left (AU)	30.74±14.03	28.23±13.92	- 8.15	NS
cc-TQ right (AU)	30.52±14.76	26.29±15.41	-13.86	NS
CBFV left (cm*sec^-1^)	41.1±7.5	42.3±8.3	+ 2.92	NS
Mean BP (mmHg)	90.65±10.33	105.06±14.52	+ 15.90	0,000
HR (beats*sec^-1^)	63.79±7.48	73.19±9.10	+ 14.74	0.000
SaO_2_	97.37±1.09	97.86±1.02	+ 0.50	0.000
End tidal CO_2_	5.0 ± 0.51	4,96 ± 0.54	- 0.70	NS
End tidal O_2_	14.95 ± 0.78	15.25 ± 0.63	+ 2.07	0.021
Respiratory rate	14.72 ± 4.30	16.08 ± 3,85	+ 9.20	0.054
MV	7.76 ± 1.52	8.97 ± 1.67	+ 15.53	0.000

^NS^ not significant; sas-TQ—the subarachnoid component of the transillumination quotient (the subarachnoid width); cc-TQ—cardiac component of transillumination quotient (pial artery pulsation); BP—blood pressure; HR—heart rate; CBFV—cerebral blood flow velocity; SaO_2_—oxyhemoglobin saturation; MV—minute ventilation; AU—arbitrary units; mm Hg—millimeters of mercury; s—seconds

CT evoked an increase in BP, sas-TQ and SaO_2_. HR, CBFV, MV, EtO_2_ and EtCO_2_ remained unchanged. A trend toward a decrease in cc-TQ was observed. The sas-TQ response was biphasic with elevation during the first 40 seconds (+8.83% versus baseline; P<0.001) and subsequent decline to +4.1% versus baseline (Table 2).

**Table 2:** Effects of 120 s CT on cc-TQ, sas-TQ, BP, HR, CBFV, EtO_2_, EtCO_2_, SaO2, respiratory rate and MV. Data presented as mean values and standard deviations (SD). All % changes are calculated with reference to baseline values.

Variable	Baseline	Cold test	% Change	P value
sas-TQ left (AU)	6023±1667	6269±1642	+ 4.10	0.021
sas-TQ right (AU)	5805±1791	5977±1828	+ 2.97	0.053
cc-TQ left (AU)	26.27±12.88	25.07±13.09	- 4.57	NS
cc-TQ right (AU)	26.56±8,64	25.09±16.02	- 5.55	NS
CBFV left (cm*sec^-1^)	40.8±8.1	41.6±7.1	+ 1.96	NS
Mean BP (mmHg)	88.26 ± 11.89	94.81±15.51	+ 7.43	0.000
HR (beats*sec^-1^)	62.85±7.06	64.30±7.17	+ 2.31	NS
SaO_2_	97.79±1.09	98.10±1.02	+ 0.32	0.022
End tidal CO_2_	4.95±0.52	5.00±0.55	+ 0.94	NS
End tidal O_2_	15.16±0.71	15.05±0.72	- 0.72	NS
Respiratory rate	15.75±3.59	16.23±4.09	+ 3.01	NS
MV	8.11±1.46	8.02±1.42	- 1.07	NS

^NS^ not significant; sas-TQ—the subarachnoid component of the transillumination quotient (the subarachnoid width); cc-TQ—cardiac component of transillumination quotient (pial artery pulsation); BP—blood pressure; HR—heart rate; CBFV—cerebral blood flow velocity; SaO2—oxyhemoglobin saturation; MV—minute ventilation; AU—arbitrary units; mm Hg—millimeters of mercury; s—seconds

**Fig 2:**
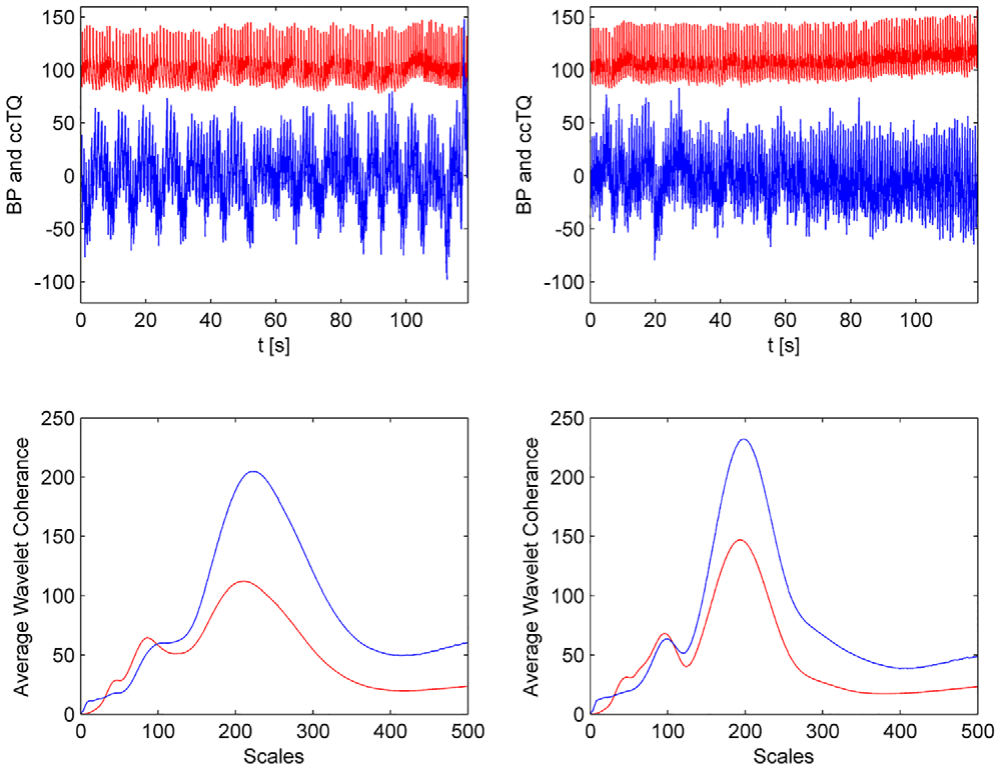
Representative tracings from 120 s baseline (left upper panel) and HGT (right upper panel) signals. The cc-TQ signal (upper panels, in blue) is less regular than the BP signal (upper panels, in red). Wavelet transform analysis reveals BP and cc-TQ peaks at ~ 1 Hz and ~ 0.3 Hz (baseline—left lower panel, HGT—right lower panel, cc-TQ analysis in blue, BP in red).

The wavelet transform analysis of the 120-s BP and cc-TQ signals is shown in Figs 2 (HGT) and 3 (CT). BP and cc-TQ peaks were observed at the human cardiac and respiratory frequencies (~1 Hz and ~0.3 Hz, respectively). No change with respect to WCO and WPCO was found between the BP and cc-TQ oscillations during HGT and CT (Table 3, Figs 4 and 5).

**Fig 3:**
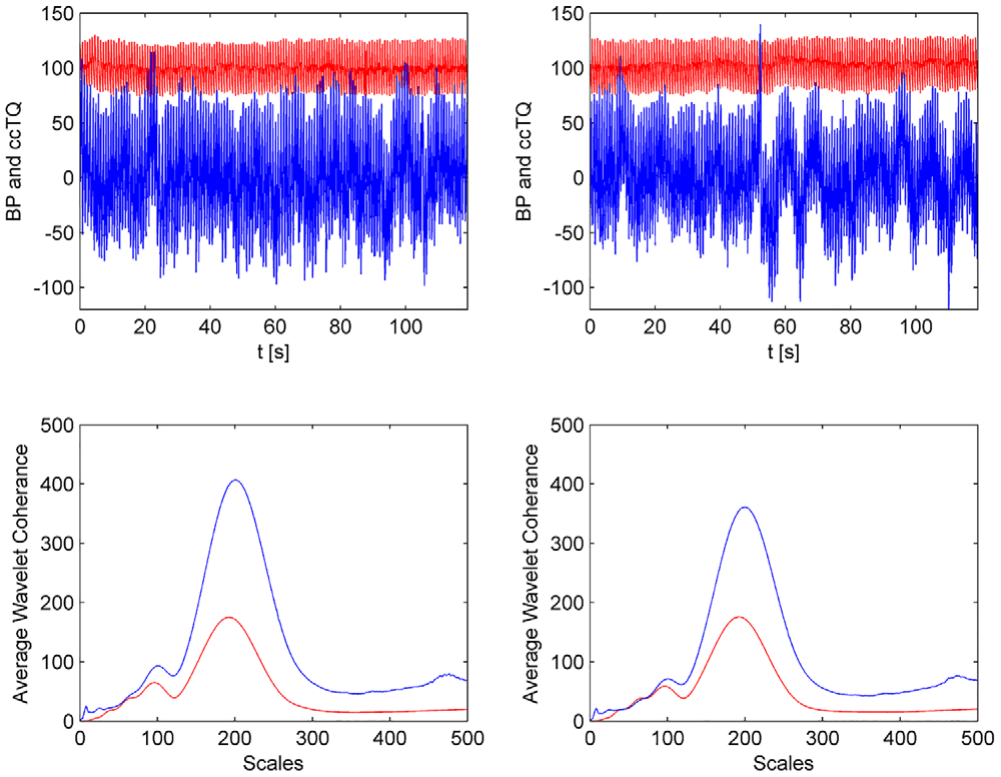
Representative tracings from 120 s baseline (left upper panel) and CT (right upper panel) signals. The cc-TQ signal (upper panels, in blue) is less regular than the BP signal (upper panels, in red). Wavelet transform analysis reveals BP and cc-TQ peaks at ~ 1 Hz and ~ 0.3 Hz (baseline—left lower panel, CT—right lower panel, cc-TQ analysis in blue, BP in red).

**Fig 4:**
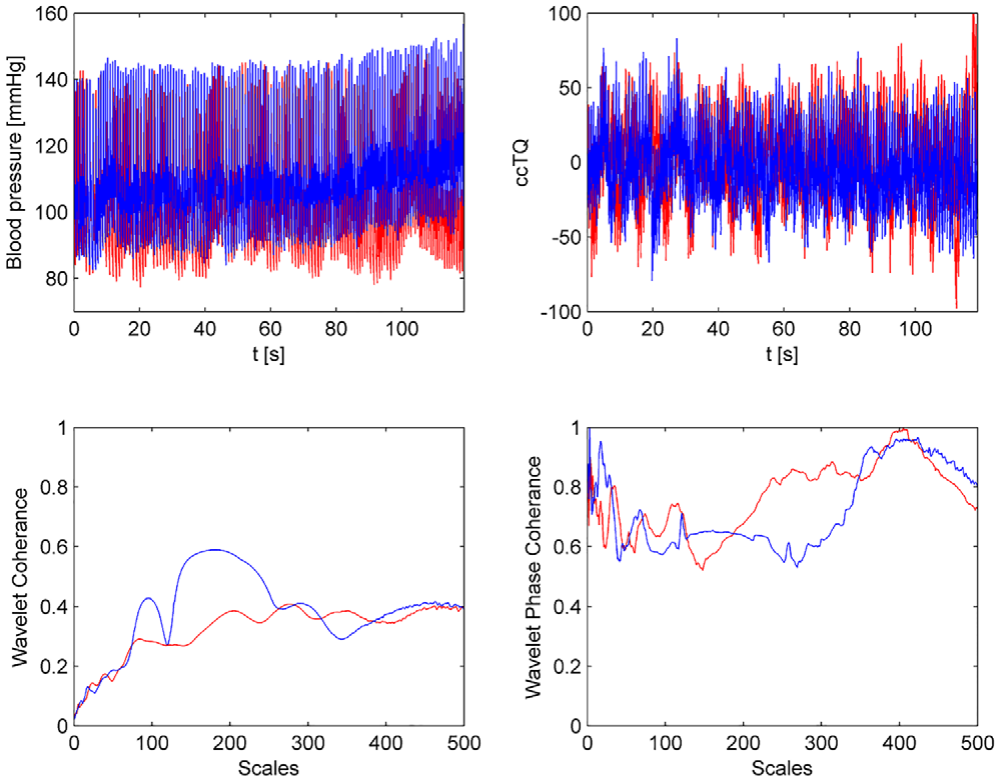
Representative tracings of signals for 120 s of baseline (in red) and HGT (in blue). BP oscillations (left upper panel), cc-TQ oscillations (right upper panel), WCO (left lower panel) and WPCO are shown (right lower panel).

**Fig 5:**
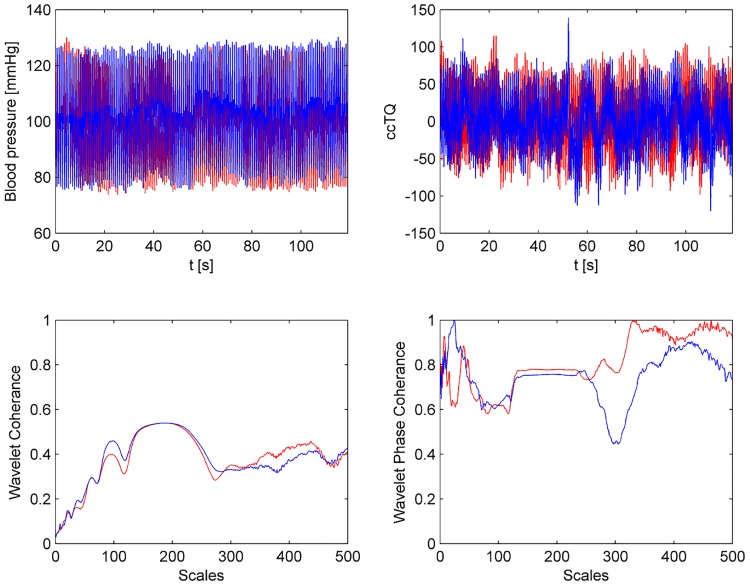
Representative tracings of signals for 120 s of baseline (in red) and CT (in blue). BP oscillations (left upper panel), cc-TQ oscillations (right upper panel), WCO (left lower panel) and WPCO are shown (right lower panel).

**Table 3:** Wavelet coherence and phase coherence between BP and cc-TQ during baseline and HGT and CT (normalized values) in left and right hemisphere.

	Cardiac frequency				Respiratory frequency			
	WCO	P	WPCO	P	WCO	P	WPCO	P
Baseline left	0.18±0.03		0.66±0.14		0.56±0.11		0.72±0.27	
HG left	0.19±0.04	0.9	0.72±0.11	0.16	0.57±0.07	0.71	0.69±0.22	0.35
Baseline right	0.21±0.11		0.66±0.18		0.53±0.09		0.74±0.24	
HG right	0.18±0.03	0.1	0.72±0.12	0.28	0.54±0.08	0.83	0.73±0.23	0.98
Baseline left	0.19±0.03		0.73±0.12		0.56±0.11		0.76±0.26	
CT left	0.19±0.02	0.94	0.71±0.13	0.47	0.53±0.05	0.32	0.77±0.22	0.92
Baseline right	0.21±0.03		0.67±0.12		0.54±0.12		0.69±0.25	
CT left	0.19±0.02	0.28	0.72±0.15	0.18	0.52±0.06	0.9	0.76±0.22	0.46

WCO—wavelet coherence; WPCO—wavelet phase coherence; left—left hemisphere; right—right hemisphere

**Fig 6:**
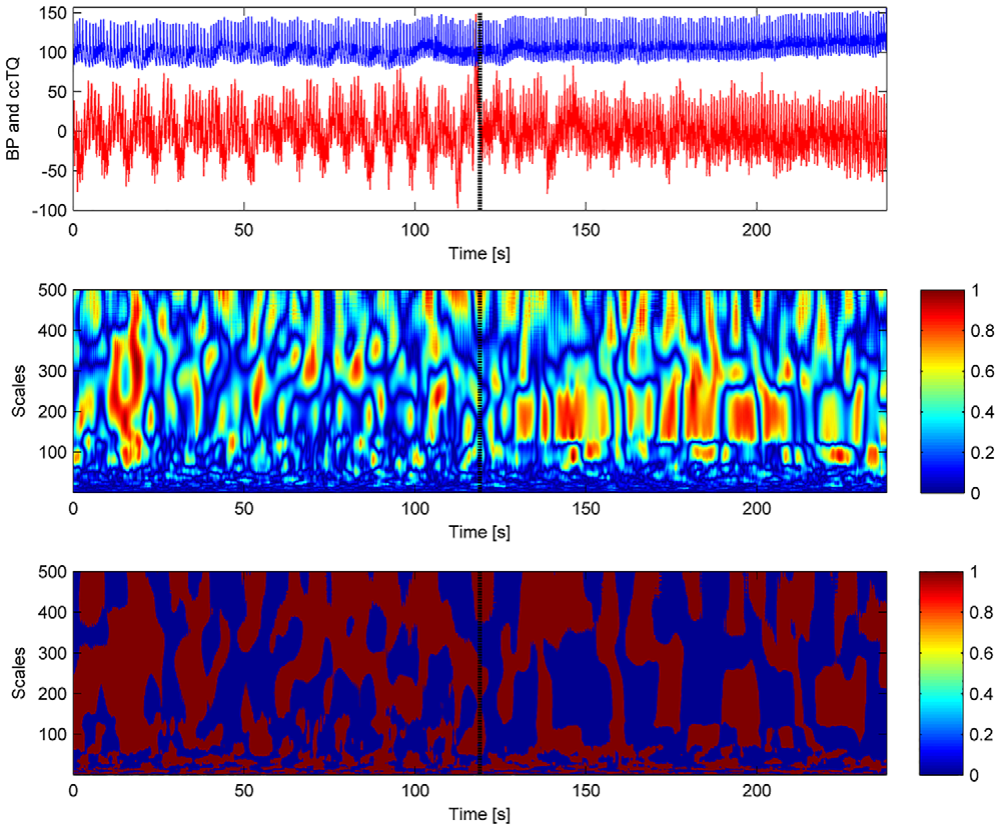
Representative WCO (middle panel) and WPCO (lower panel) tracings. BP (red) and cc-TQ (blue) signals are provided in the upper panel. WCO and WPCO remains relatively stable throughout baseline and HGT. Peak values at ~1 Hz and ~0.3 Hz are visible. The black marker indicates the start of HGT.

**Fig 7:**
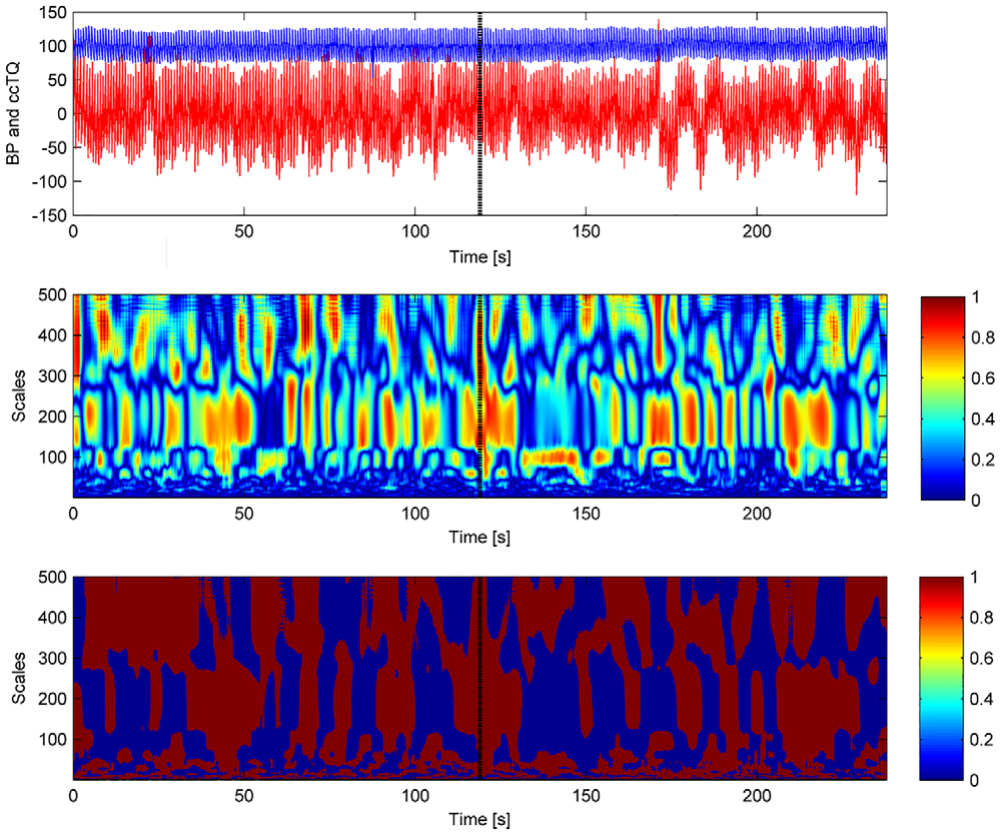
Representative WCO (middle panel) and WPCO (lower panel) tracings. BP (red) and cc-TQ (blue) signals are provided in the upper panel. WCO and WPCO remains relatively stable throughout baseline and CT. Peak values at ~1 Hz and ~0.3 Hz are visible. The black marker indicates the start of CT.

The time-courses of WCO and WPCO during HGT and CT are shown in Figs 6 and 7, respectively. No significant differences were noted between the left and right hemispheres with respect to the analysed variables. We did not observe any differences due to sex.

## Discussion

There were two main findings of the study in healthy subjects: 1) short sympathetic activation does not affect the cardiac and respiratory contribution to the relationship between BP—pial artery pulsation oscillations, 2) HGT and CT display divergent effects on the width of the subarachnoid space, an indirect marker of changes in intracranial pressure.

The role of SNS in CBF regulation remains a matter of controversy [22–24]. Nevertheless, evidence has accumulated that sympathetic neural control of the CBF may exert a protective effect, particularly during increases in cerebral perfusion pressure due to sudden BP elevations [25–28]. In this study, we assessed the indirect SNS effect on CBF. The cardiac role in CBF maintenance is increasingly recognised [4,10,18,29–31]. Furthermore, the contribution of the lung to brain homeostasis in health and disease has recently been highlighted [32–34]. This study clearly suggests that short SNS activation does not affect the cardiac and respiratory contribution to the relationship between BP—pial artery pulsation oscillations in healthy subjects. Our finding is further strengthened by the fact that long apnoea’s performed by elite breath-hold divers also do not affect the cardiac contribution to the relationship between BP and pial artery pulsation oscillations, at least when using wavelet transform analysis [35]. The prolonged apnoea in elite breath-hold divers is associated with an enormous increase in SNS activity [36]. Both HGT and CT evoked increases in HR and BP. In addition, during HGT, an increase in respiratory rate and MV was observed. In spite of these changes, WCO and WPCO between BP and pial artery pulsation remained constant. We can speculate that stabilisation of the cardiac contribution to the BP/cc-TQ relationship may represent another mechanism by which the integrity of brain vessels is protected during sudden BP increases.

Alternatively, we are tempted to consider that SNS mediated CBF control mechanism is biphasic. Pial artery time to response to BP fluctuations is limited to seconds [14,28,31] and central CBF control seems to be quicker than peripheral autonomic mechanisms aiming at stabilisation of CBF [19, 37]. Relatively short apnoea in normal subjects lead to decrease in cardiac contribution to the relationship between BP and pial artery pulsation oscillations [18]. At the same time much longer apnoea performed by elite apnoea divers and associated with extreme elevation of SNS activity [36] do not affect wavelet coherence between BP and cc-TQ signals at the cardiac frequency [35]. Therefore, we can assume that as long as CBF is controlled over by central regulatory mechanisms cardiac contribution to the relationship between BP—pial artery pulsation oscillations is not affected (like in this study) or driven by parasympathetic system to maintain oxygen supply/demand balance in the heart (for example, during short apnoea) [18]. However, in case of extreme hypoxia cardiac contribution to the BP—pial artery pulsation oscillations relationship is stabilised by the SNS to maintain cerebral oxygenation even at the expense of potential oxygen supply/demand mismatch in the heart [35].

We decided to examine the whole duration of HGT and CT (120 s). These periods were compared to the 120 s at baseline (Figs 2, 3, 4, 5, 6 and 7). We used wavelet transform analysis as it provides windows of adjustable lengths, thereby providing the benefit of showing high resolution at cardiac and respiratory frequencies. Compared with autoregressive estimation, wavelet transform is calculated directly from data, and the limitations of linear modelling and the choice of model order are thus avoided [38]. The method has already been used by us and others [10,18,21,38–40]. In finite-length signals, less variation occurs in the phase difference if fewer periods are analysed, and this may result in artificially increased phase coherence. Usually, to identify a point that demarcates truly significant coherence in fewer periods, surrogate analysis amplitude-adjusted Fourier transform is used. Surrogate signals are generated by shuffling the phases of the original time series to create new time series with the same means, variances and autocorrelation functions (and therefore, the same power spectra) as the original sequences, but without their phase relations [41]. In this study, however, we analysed only high-frequency parts of the signal (~1 Hz and ~0.3 Hz) from a relatively long data series (120 s), and therefore we believe that use of surrogate analysis amplitude-adjusted Fourier transform was not needed. Our methodology to analyse WCO and WPCO at human cardiac frequency was described previously [18].

It has been shown by our team and others that HGT increases cerebral blood volume and decreases the subarachnoid width, suggesting an increase in intracranial pressure [28,42]. In contrast, Wilson and colleagues [43] suggested that the CT may selectively decrease cerebral blood volume in grey matter. In Wilson’s study, the CT was performed in a supine position for approximately 2 minutes, and the contrast-enhanced computed tomography scans occurred between the 60th and 90th seconds [43]. Therefore, the experimental conditions in Wilson’s study were very similar to our design. Our results are in accordance with Wilson’s results, as a slight increase in the sas-TQ indicates a decline in the cerebral blood volume and intracranial pressure during CT. Wilson proposed that regulation of cerebral blood volume is maintained through a direct influence of the SNS on cerebral veins, with SNS activation leading to venoconstriction [43]. In this study, we have shown for the first time in the same group of healthy subjects that HGT and CT display divergent effects on the width of the subarachnoid space, an indirect marker of intracranial pressure. HGT and CT are transmitted by different central SNS circuits [15–17]. Taken together, it seems that—regardless of the mechanism—the influence of SNS activation on cerebral blood volume and intracranial pressure depends on which central SNS pathways are involved.

The high within- and between-subject reproducibility and repeatability of NIR-T/BSS measurements were demonstrated previously [11]. NIR-T/BSS, like NIRS, allows for direct within-subject comparisons [11,44]. As long as changes from baseline values are analysed, high between-subject reproducibility is observed. However, measurements with the use of IR light (NIRS and NIR-T/BSS) do not allow for direct comparisons between subjects due to differences in skull bone parameters [11,44]. We did not observe any sex related differences. Nevertheless, it should be noticed that the study was not aimed at, and not powered to detect such differences.

NIR-T/BSS offers unique possibility to measure non-invasively direction of changes in ICP in diseases like stroke, traumatic brain injury or obstructive sleep apnoea. The “standard of care” monitoring of ICP is highly invasive and therefore restricted to critically ill patients or post neurosurgery procedures. Non-invasive, even if indirect, monitoring of ICP opens completely new fields in cardiovascular and neurological research. Exposure to pulsatile pressure and augmented flow, which exists in the carotid artery may lead to vascular damage [45] and/or produce hypertrophy of cerebral arterioles, even in the absence of increases in mean BP [46]. Therefore, better understanding of the relationship between BP and pial artery pulsation may create new ways in management of diseases of high societal impact like stroke or vascular dementia. We believe that combination of NIR-T/BSS with advanced signal analysis tools most likely represents a promising approach in describing the interrelations and pathways involved in stroke, vascular dementia and other cerebrovascular diseases. The presented results establish therefore reference for future clinical studies which are warranted.

To conclude, we have shown that SNS activation does not affect the cardiac and respiratory contribution to the relationship between the BP and pial artery pulsation oscillations in healthy subjects. In fact, it seems that a high sympathetic drive tends to stabilise the relationship between the analysed signals. The current study establishes the reference for future research in subjects with autonomic imbalances, for instance, in subjects suffering from obstructive sleep apnoea. Furthermore, we have demonstrated that HGT and CT exert opposite effects on the subarachnoid width, an indirect marker of intracranial pressure. We postulate that the SNS influence on cerebral blood volume and regulation of intracranial pressure may vary depending on the involvement of particular central SNS circuits.
